# Neonicotinoid and s‐triazine pesticide transport dynamics in a small karst agricultural watershed

**DOI:** 10.1002/jeq2.70155

**Published:** 2026-02-23

**Authors:** Henry J. Kibuye, Tamie L. Veith, Tyler A. Groh, Heather E. Preisendanz

**Affiliations:** ^1^ Department of Agricultural & Biological Engineering The Pennsylvania State University University Park Pennsylvania USA; ^2^ USDA‐ARS, Pasture Systems and Watershed Management Research Unit University Park Pennsylvania USA; ^3^ Department of Ecosystem Science and Management The Pennsylvania State University University Park Pennsylvania USA; ^4^ Institute for Sustainable Agricultural, Food, and Environmental Science, College of Agricultural Sciences The Pennsylvania State University University Park Pennsylvania USA

## Abstract

The potential ecological and human health risks posed by agricultural pesticides necessitate a comprehensive understanding of pesticide transport dynamics to guide effective management. Riparian buffers are often implemented to mitigate nutrients and sediment transported in surface runoff. However, the co‐benefits they may offer for pesticide mitigation are not well understood, especially in karst landscapes. We monitored nested catchments in a 62 km^2^, karstic, agricultural watershed in the siliciclastic Appalachian Mountain physiographic province of the eastern United States to assess transport dynamics of two surface‐applied s‐triazine herbicides (atrazine and simazine) and four neonicotinoids commonly coated on seeds (clothianidin, imidacloprid, thiacloprid, and thiamethoxam). In‐stream grab samples were collected biweekly from five sites during the 2023 growing season. Simazine, atrazine, and clothianidin were the most frequently detected compounds, found in 93%, 92%, and 75% of samples collected, respectively. Concentration‐discharge relationships indicated that clothianidin and atrazine were mobilized in surface runoff and could be retained in buffers. However, simazine appeared to be transported primarily via groundwater from application site to stream through karst features within the fields, making it less likely to be mitigated by riparian buffers. Pesticide fluxes at upstream sub‐watersheds were found to be positively correlated to downstream fluxes due to hydrological connectivity typical of head watersheds, agricultural land use distributed across the entire watershed, and the influence of karst features. These patterns suggest the importance of enhancing the common approach of targeting “hot spot” sub‐watersheds based on surface hydrology with consideration of subsurface transport pathways that may cross sub‐watershed boundaries.

## Core Ideas

- Stream sampling most often detected surface‐applied s‐triazines and highly water‐soluble clothianidin.
- Karst hydrology enables upstream applications to transport downstream with minimal dilution.
- Local buffers may not capture contaminated karst flow that discharges to streams through streambank seeps.
- Atrazine and clothianidin were transported in surface runoff and could likely be mitigated by riparian buffers.
- Surface‐applied simazine was largely transported in groundwater, likely bypassing riparian buffer treatment.

## Plain Language Summary

Vegetative buffers protect streams from contaminants transported by surface runoff. Surface‐applied and seed‐coated pesticides can be transported to streams through surface and groundwater. The extent to which buffers may mitigate pesticides is unclear, particularly in karst watersheds. We sampled five locations in a karst agricultural watershed during the 2023 growing season. Samples were analyzed for two surface‐applied pesticides and four seed‐coated pesticides. How concentrations varied with flowrate was used to identify transport pathways. We found that two pesticides (one surface‐applied and one seed coated) were mobilized in surface runoff, suggesting that buffers could help mitigate them. However, one surface‐applied pesticide moved through groundwater, likely bypassing buffer treatment. The remaining pesticides showed no dominant transport pathways. This study highlights the need to consider transport pathways to mitigate pesticide transport, particularly in karst watersheds.

**Abbreviations**
C‐Q: concentration‐discharge
CV: coefficient of variation
MDL: method detection limit

## 1 INTRODUCTION

Different classes of pesticides are currently used in agriculture to increase yields and produce crops that are free of pests and disease‐causing pathogens. However, during and following pesticide application, these compounds can be mobilized through volatilization, leaching, and/or surface runoff (Alsafran et al., 2022; Aydinalp & Porca, 2004; Tudi et al., 2021). Unless trapped or neutralized, these compounds and their metabolites eventually reach waterbodies where they can affect non‐target aquatic species and lead to adverse human health impacts in populations reliant on the impaired water resources (Ahmad et al., 2024; Ansari et al., 2021; Levengood & Beasley, 2007; Syafrudin et al., 2021; Williams & Sweetman, 2019).

Despite usage standards and policies, monitoring studies have documented pesticide occurrences in streams, both in urban and agricultural settings, and sometimes at concentrations above aquatic life benchmarks (Ensminger et al., 2013; Stone et al., 2014; Sullivan et al., 2009). Further, investigations of pesticide transport pathways have indicated an interplay of pesticide use patterns, pesticide physical and chemical properties, environmental and geologic conditions, and hydrological factors that influence their persistence and occurrence away from points of use (Bexfield et al., 2021; Reedich et al., 2017; Reh et al., 2013; Scribner et al., 2005; Stackpoole et al., 2021; Wolfram et al., 2018).

Studies have indicated that conservation practices typically implemented to meet sediment and nutrient reduction goals, such as riparian buffers, can offer co‐benefits in mitigating pesticide transport. A review by Krutz et al. (2005) found that in all but two cases, vegetative filter strips reduced herbicide transport from source areas by at least 27%, while a plot‐scale study by Lerch et al. (2017) showed that vegetative buffer strips were effective in reducing herbicide loads by at least 19%. Arora et al. (2010) reported that agricultural buffer strips had the potential of retaining sediment‐bound pesticides in runoff by an average range of 61%–76%. Variation in retention is influenced by the physicochemical properties of the pesticides, as well as spatially varying factors, including environmental conditions, the extents, and characteristics of implemented mitigation practices (Boyd et al., 2003; Chow et al., 2020; Lerch et al., 2017; Ryberg & Gilliom, 2015; Vormeier et al., 2023).

In regions with karst topography, rapid transport of some pesticides to groundwater and others through overland flow (Huang et al., 2021; Schorr et al., 2024) makes it challenging to identify field‐edge or riparian areas to target for effective restoration. Additionally, these transport dynamics hinder the assessment of the effectiveness of existing agricultural conservation practices implemented to reduce nutrient and sediment transport in also mitigating pesticides. Factors such as precipitation, topographical factors (e.g., karst features), and concentration‐discharge (C‐Q) relationships must be considered. Therefore, monitoring at the sub‐watershed scale, in addition to the watershed outlet, and understanding both surface and subsurface hydrology throughout the watershed is critical to understanding pesticide transport dynamics and identifying gaps or opportunities in contaminant control practices (Chow et al., 2020; Do et al., 2012; Jiang et al., 2020; Lookenbill & Arnold, 2023).

The objectives of this study were to examine the transport dynamics of surface‐ and subsurface‐applied agricultural pesticides in a karst watershed and to assess the potential for riparian buffers, typically implemented to mitigate sediment and nutrient loadings, to also mitigate pesticide transport. Specifically, we aimed to (i) quantify the extent to which pesticides are transported via surface runoff and therefore likely to be mitigated by riparian buffers and (ii) assess how karst features may influence transport pathways and implications for the effectiveness of conventional “hot spot” targeting of conservation practice implementation. To address these objectives, we quantified the occurrence patterns and fluxes of neonicotinoid insecticides and s‐triazine herbicides at five nested monitoring sites within a small agricultural head watershed and characterized the influence of hydrology (precipitation and streamflow dynamics) and karst features on pesticide transport. This study provides a better understanding of the factors affecting pesticide transport in a small karst agricultural watershed and provides a basis for evaluating potential co‐benefits of sediment‐ and nutrient‐focused conservation practices for pesticide mitigation.

## 2 MATERIALS AND METHODS

### 2.1 Study area

**FIGURE 1:**
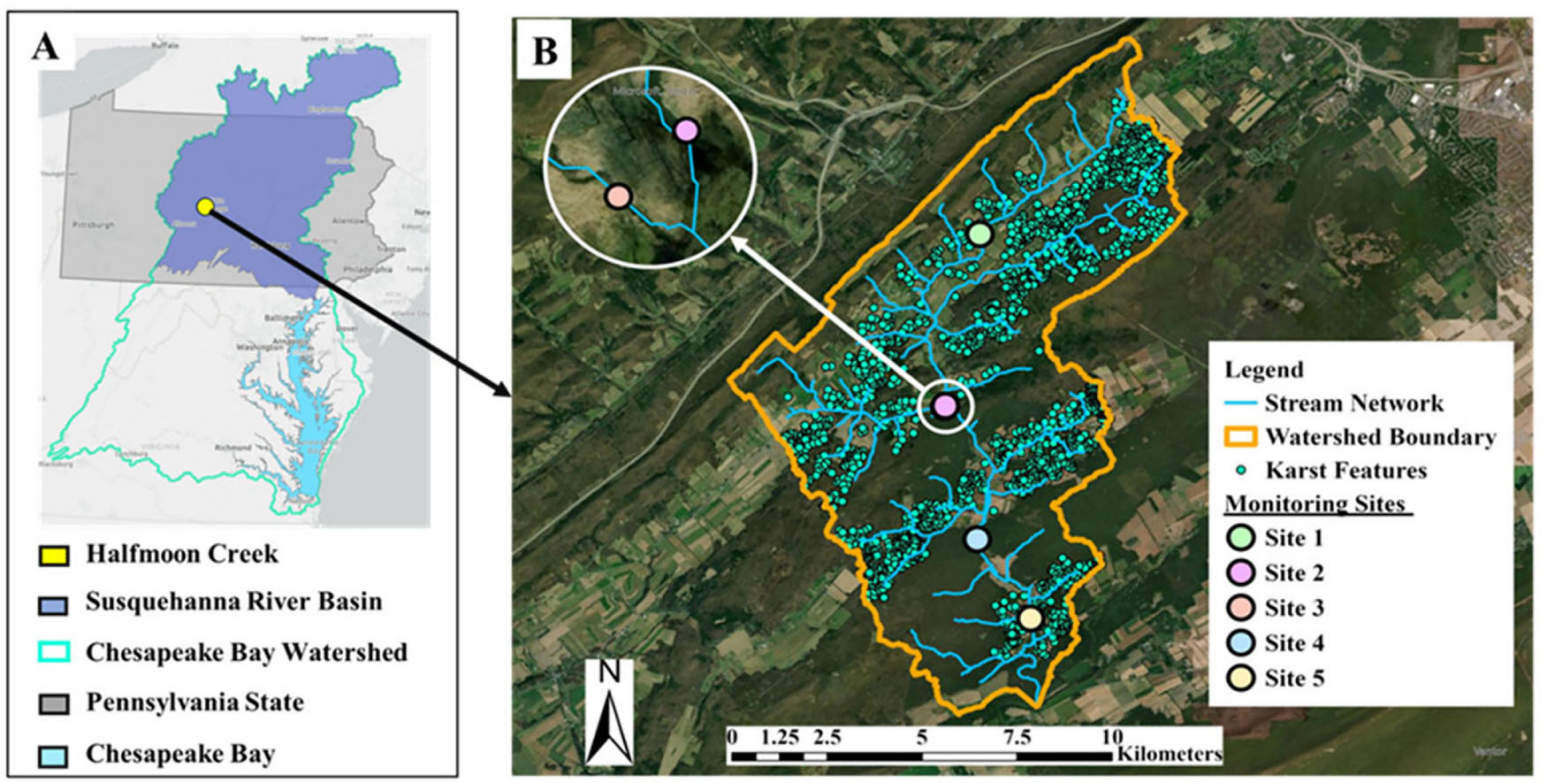
(A) Location of the Halfmoon Creek Watershed within the Chesapeake Bay Watershed in eastern United States; and (B) locations of karst features across the watershed identified using aerial imagery, questionnaires, and historical publications (PASDA, 2007), and the five water quality monitoring sites.

This study was conducted in the Halfmoon Creek watershed in central Pennsylvania (Figure 1). The watershed (US hydrologic unit code 020503020402) spans 62 km^2^ within the headwaters of the Lower Susquehanna River Basin, draining to the Chesapeake Bay (Figure 1). The climate is humid continental, with daily and seasonal temperature variations, high precipitation, warm summers, and cold winters. The major soil series in the watershed are Morrison, Hublersburg, and Hagerstown (Table S1), with sandy loam, silt loam, and channery silt loam as the main textures (NRCS, 2024). The watershed is located within the siliciclastic Appalachian Mountain physiographic province, with narrow ridges and wide fertile valleys of limestone, sandstone, and dolomite (Figure 1; Table S2). These karstic landscapes are often characterized by caves, underground streams, springs, surface depressions, and sinkholes. They are a concern to surface and groundwater quality due to rapid infiltration of contaminants through the fissures into underground aquifers and translocation from the source of contamination (Huang et al., 2021; Selak et al., 2022). Across the watershed, karst features (Figure 1) include 97% surface depressions, 2.5% sinkholes, and < 1% caves, with 40% of the features intersecting agricultural fields.

Halfmoon Creek, a designated high‐quality cold‐water fishery (25 Pa. Code § 93.9n., 2025), can support diverse benthic macroinvertebrates and cold‐water fish species, such as trout. It is also a popular destination for fly‐fishing activities. However, the watershed is currently impaired by siltation, necessitating the establishment of a community‐supported, sediment‐focused watershed restoration plan (Chesapeake Bay Foundation, 2021; Pennsylvania DEP, 2018). Five stream sites within nested sub‐watersheds were selected for hydrologic and water quality monitoring (Figure 1) in conjunction with the other activities of the restoration plan.

**Core Ideas**

- Stream sampling most often detected surface‐applied s‐triazines and highly water‐soluble clothianidin.
- Karst hydrology enables upstream applications to transport downstream with minimal dilution.
- Local buffers may not capture contaminated karst flow that discharges to streams through streambank seeps.
- Atrazine and clothianidin were transported in surface runoff and could likely be mitigated by riparian buffers.
- Surface‐applied simazine was largely transported in groundwater, likely bypassing riparian buffer treatment.

### 2.2 Sub‐watershed land use

**FIGURE 2:**
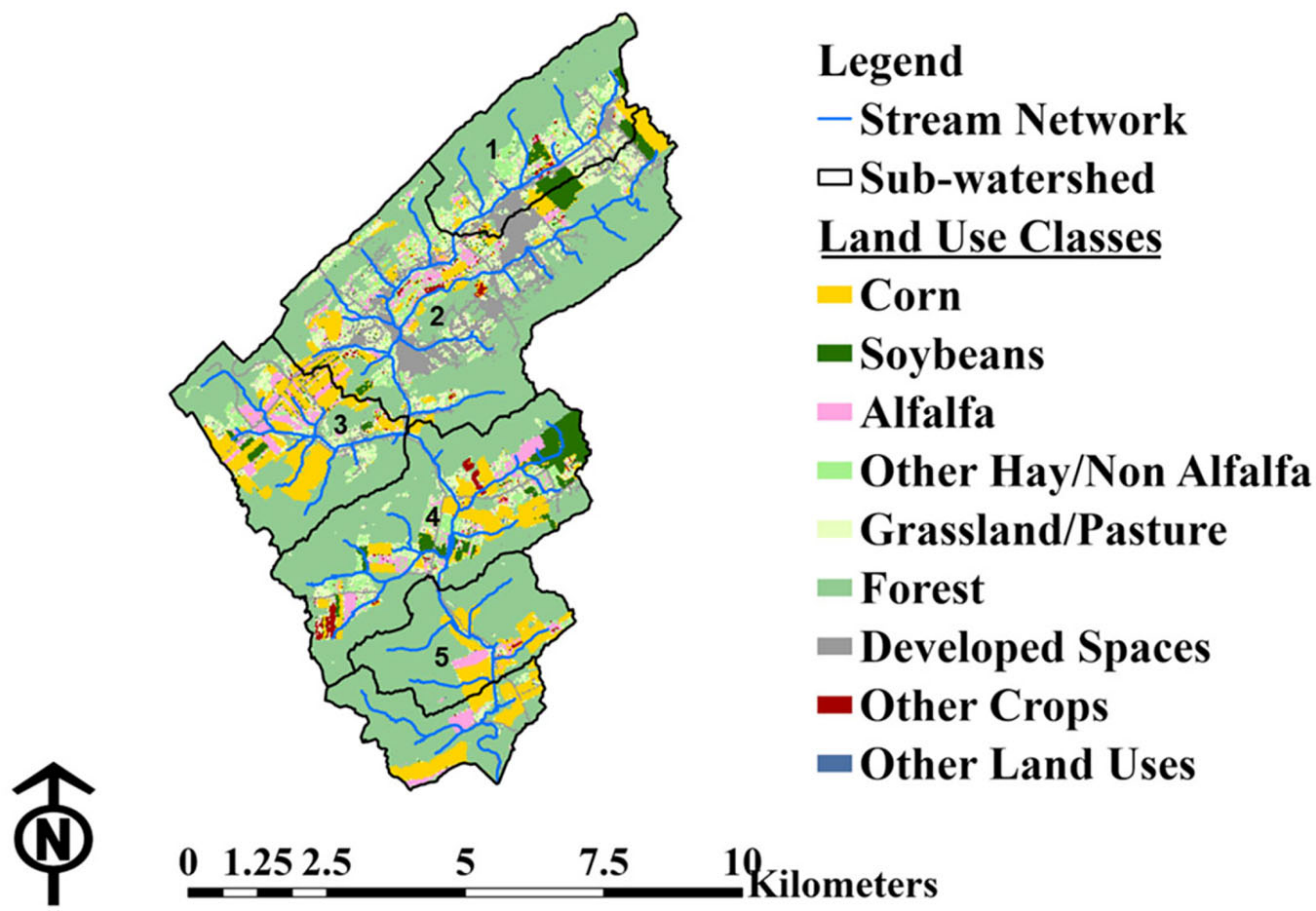
The 2023 land use‐land cover distributions for each monitoring site sub‐watershed (USDA National Agricultural Statistics Service, 2024).

Surface sub‐watersheds for the monitoring sites were delineated in ArcGIS Pro version 3.1.0 with 10‐m elevation maps. (ESRI, 2023; U.S. Geological Survey, 2022). Land use maps were created using the 30‐m USDA‐NASS crop data layer for 2023 (USDA National Agricultural Statistics Service, 2024), which distinguishes crop types within the cropland areas (Figure 2). The local land use within each site's sub‐watershed and cumulative percentage of each land use class in all upstream sub‐watersheds was then calculated for each monitoring site (Table 1). These data were used to inform the influence of upstream land uses on downstream water quality. Further, land use percentages within the riparian buffer zones (18 m on each side of the stream) for each monitoring site sub‐watershed were determined (Table 1). Note that 18 m is the recommended pesticide application setback distance in this region, within which conservation management implementation is recommended to meet water quality goals (7 Pa. Code § 128.103, 2025; Brittingham & DeCecco, 2024; Vormeier et al., 2023).

**TABLE 1:** Local and cumulative upstream percentages (%) of major land cover classes of each monitoring site sub‐watershed (top) and within the 18‐m riparian zones of both sides of the streams in each sub‐watershed (bottom).

	Site 1	Site 2		Site 3	Site 4		Site 5	
	Local	Local	+Upstream	Local	Local	+Upstream	Local	+Upstream
**Sub‐watershed**								
Corn	4.0	7.4	6.5	22.2	10.9	10.5	15.5	11.0
Soybeans	5.2	2.3	3.1	2.1	8.0	4.2	0.0	3.8
Alfalfa	2.0	4.0	3.4	7.8	5.0	4.6	3.3	4.5
Hay	8.6	6.1	6.8	4.1	6.7	6.3	0.9	5.7
Grassland/pasture	17.3	12.8	14.1	9.4	8.4	11.7	2.3	10.6
Forest	52.4	50.4	51.0	48.5	55.6	51.8	76.0	54.5
Urban/residential	9.7	16.1	14.3	5.7	3.7	9.9	1.6	9.0
Other crops	0.7	0.7	0.7	0.2	1.8	0.9	0.3	0.9
Other land uses	0.1	0.1	0.1	0.0	0.0	0.1	0.0	0.1
**Riparian zone**								
Corn	1.4	4.6	3.7	19.0	13.0	8.9	25.9	10.9
Soybeans	0.2	0.8	0.7	1.0	15.3	4.6	0.0	4.1
Alfalfa	2.8	2.2	2.4	5.7	9.1	4.7	2.5	4.5
Hay	11.8	8.1	9.2	4.7	12.9	9.4	0.8	8.4
Grassland/pasture	34.7	23.3	26.5	25.2	16.0	23.4	12.1	22.1
Forest	40.8	38.8	39.3	29.7	25.2	33.8	54.3	36.2
Urban/residential	7.3	20.9	17.1	14.5	6.9	13.9	4.1	12.8
Other crops	0.7	1.2	1.0	0.2	1.6	1.1	0.3	1.0
Other land uses	0.2	0.1	0.1	0.0	0.1	0.1	0.0	0.1

### 2.3 Hydrological monitoring

Between 2019 and 2024, stream cross‐section measurements were taken periodically within 500 m of the monitoring sites to calculate discharge using the mid‐section method (Turnipseed & Sauer, 2010) and develop rating curves. Average water level was recorded in 15‐min intervals with a Campbell Scientific stainless steel pressure transducer (CS‐451) connected to a Campbell Scientific datalogger (CS‐1000, CS‐1000X, or CS‐850). During each sampling event, stage measurements at the center of the stream cross‐sections were manually taken to validate the sensor measurements and enable corrections, if needed. These measurements were utilized to estimate discharge at the time of sampling using the rating curves. The discharge values at the time of sampling spanned over 90% of the flow duration curve developed using the 15‐min measurements taken for the period of study, indicating that sampling captured nearly all flow conditions (Figure S1). Precipitation data were obtained from a private weather station within the watershed listed on Weather Underground as KPAPORTM52 (situated within Site #2's sub‐watershed, approximately 40.78°N, −78.01°W). Precipitation was recorded using AccuWeather WS‐2000 Ambient Weather System with an accuracy of ± 10% and resolution of 0.254 mm.

### 2.4 Sample collection and processing

Biweekly grab water samples were collected during the crop growing season, from mid‐April to the end of October 2023, using 1‐L trace‐cleaned amber glass bottles. This sampling regime was designed to capture pesticide dynamics during the growing season, including the time of planting and pesticide applications (around April 29) through harvest (around October 12) (Chesapeake Bay Foundation, 2021). One field blank sample was collected during each sampling event, with deionized water in a 1‐L amber glass bottle opened at each site while grab sampling was conducted. Samples were transported on ice to the Penn State Environmental Contaminants Analytical Laboratory, processed within 72 h (see Supporting Information), and analyzed following US EPA Method 1694 (US EPA, 2007).

The samples were analyzed for two s‐triazine herbicides: atrazine and simazine; and four neonicotinoids: clothianidin, imidacloprid, thiacloprid, and thiamethoxam (Table 2). These pesticides are widely used in agricultural and urban settings. Atrazine and simazine are surface‐applied as pre‐ and post‐emergence herbicides to control perennial grass and annual broadleaf weeds in agricultural fields, including corn (LeBaron et al., 2008). Neonicotinoids are a group of systemic insecticides used to control a wide variety of sucking pests (Jeschke et al., 2011). As seed dressings, clothianidin is predominantly used in corn, imidacloprid in soybean, and thiamethoxam in both corn and soybeans. (Douglas & Tooker, 2015; Jeschke et al., 2011; Krupke et al., 2012; US EPA, 2003, 2014; USGS, 2024). Thiacloprid is used primarily in specialty crops as foliar sprays.

**TABLE 2:** Physicochemical properties, method recoveries, and method detection limits (MDL) of the target pesticides.

	Clothianidin	Imidacloprid	Thiacloprid	Thiamethoxam	Atrazine	Simazine
**Solubility (mg/L)** ^a^	327	610	184	4100	35	5
**Hydrophobicity log *K*_ow_** ^a^	0.905	0.57	1.26	−0.13	2.7	2.3
**Soil affinity log *K*_oc_** ^b^	1.78	2.19–2.90	3.04	1.84	1.41–3.07	1.89–3.55
**Chronic toxicity (ng/L)** ^c^	50	10	970	740	60,000	40,000
**Average recovery (%)**	73	74	75	85	35	43
**Method detection limit (MDL) (ng/L)**	1	1	0.4	1	0.4	0.4

^a^ Solubility in water (mg/L) and octanol‐water partitioning coefficient (Kow) values at 20°C and at pH 7. Data source: https://sitem.herts.ac.uk/aeru/iupac/

^b^ Soil organic carbon partitioning coefficient (Koc) values. Data source: https://pubchem.ncbi.nlm.nih.gov/

^c^ US EPA. Aquatic Life Benchmarks and Ecological Risk Assessments for Registered Pesticides. Data source: https://www.epa.gov/pesticide‐science‐and‐assessing‐pesticide‐risks/aquatic‐life‐benchmarks‐and‐ecological‐risk. Aquatic life benchmark.

### 2.5 Concentration and discharge coefficient of variation

To investigate pesticide transport dynamics over the sampling period, coefficients of variation for pesticide concentration (CV_C_) and discharge (CV_Q_) were computed. Only concentrations above the respective method detection limits (MDLs) (Table 2) were used in the CV_C_ calculation. Thompson et al. (2011) established a CV_C_/CV_Q_ threshold of 0.3, with ratios <0.3 and >0.3 implying chemostatic and episodic transport dynamics of the contaminant, respectively. Other previous studies have used the CV_C/_CV_Q_ ratio to assess transport dynamics of agricultural chemicals and contaminants of emerging concern (Gall et al., 2015; Kibuye et al., 2020).

### 2.6 Quantifying concentration discharge relationship

To further understand pesticide transport dynamics, C‐Q relationships were assessed for each of the pesticides and at each of the monitoring sites. The C‐Q relationship is given by the empirical power‐law relationship *C = aQ^b^*, with *a* and *b* as constants. The relationship can be linearized by plotting C versus Q on a log‐log scale, with *b* calculated as the slope of the line. The slope (*b*) indicates how concentration changes as a function of discharge, with studies reporting *b* < 0, *b* > 0, and *b = *0 as representing dilution, accretion, and chemostatic transport dynamics, respectively (Gall et al., 2015; Knapp et al., 2020; Musolff et al., 2015; Vogel et al., 2005). Similar to the CV_C_ calculation above, only pesticide concentrations above the MDL were included in the analysis.

### 2.7 Data analysis

The influence of precipitation on pesticide concentration across the monitoring period and at seasonal levels was assessed with Pearson correlation, using cumulative 3‐day precipitation prior to each sampling event and mean pesticide concentration across sampling sites for each sampling event. The coefficient (*r*) was used to examine the strength and direction of the correlation alongside the corresponding *p*‐value. Pesticide mass flux into the stream was calculated by multiplying the concentration by the area‐normalized discharge during the respective sampling event (Stoeckel et al., 2012). A concentration value of $\mathrm{MDL}/\sqrt{2}$ was used when analyzed values were below the MDL (Antweiler & Taylor, 2008; Gall et al., 2015).

A one‐sided Wilcoxon signed‐rank test was performed to determine whether downstream pesticide fluxes were significantly greater than their immediate upstream fluxes using paired concentrations from each sampling event. Further, the non‐parametric Spearman's rank correlation coefficient (*ρ*) was calculated along with the associated *p*‐value to assess how strongly upstream and downstream fluxes varied together and the level of significance. The downstream sites were Site #2 (downstream of Site #1), Site #4 (downstream of Sites #2 and 3), and Site #5 (downstream of Site #4) (see Figure 1). Statistical analysis was performed in RStudio version 4.3.1 using base R “stats” package and functions (R Core Team, 2023), data manipulation using “tidyr” and “dplyr” (Wickham, François, et al., 2023; Wickham, Vaughan, et al., 2023), and visualization using “ggplot2” (Wickham, 2016).

## 3 RESULTS AND DISCUSSION

### 3.1 Summary of pesticide occurrence

#### 3.1.1 s‐Triazine detection frequencies and concentrations

Atrazine and simazine were detected most frequently during the study period, present in at least 86% and 93% of the samples collected at each site, respectively (Table 3). Atrazine was detected at concentrations ranging from 4.1 to 160 ng/L. High concentrations of atrazine during the spring led to high standard deviations in atrazine concentrations at all sites except Site #1 (Table 3). The mean seasonal concentrations across all sites were 56.7, 16.3, and 9.1 ng/L (Table S3) in spring, summer, and early fall, respectively. Simazine was detected at concentrations ranging from 0.97 to 4.7 ng/L. Mean seasonal concentrations across sites exhibited little variability, with values of 2.9, 2.5, and 2.6 ng/L (Table S3), respectively, in spring, summer, and early fall.

**TABLE 3:** Summary of frequency of detections (FoD) above the method detection limit (MDL), mean concentration ± standard deviation (SD), and maximum (max) concentrations (ng/L) of pesticides at each monitoring site.

	Atrazine			Simazine			Clothianidin			Imidacloprid			Thiamethoxam		
Site	FoD (%)	Mean ± SD	Max	FoD (%)	Mean ± SD	Max	FoD (%)	Mean ± SD	Max	FoD (%)	Mean ± SD	Max	FoD (%)	Mean ± SD	Max
**1**	93.3	9.66 ± 3.31	15	93.3	2.47 ± 0.92	4	33.3	1.28 ± 0.25	1.7	6.7	NA	1.1	0.0	NA	–
**2**	93.3	27.92 ± 37.28	140	93.3	2.24 ± 0.72	3.3	80	2.18 ± 0.83	4	20.0	4.23 ± 4.65	9.6	6.7	NA	1.9
**3**	93.3	32.15 ± 35.01	140	93.3	3.63 ± 0.93	4.7	93.3	6.73 ± 6.31	26	6.7	NA	1.1	0.0	NA	–
**4**	86.7	26.78 ± 34.81	130	93.3	2.51 ± 0.53	3.5	80	3.26 ± 1.86	8.2	20.0	1.7 ± 0.72	2.5	0.0	NA	–
**5**	93.3	31.42 ± 42.61	160	93.3	2.34 ± 0.67	3.3	86.7	3.34 ± 2.05	9.3	13.3	2.5 ± 1.41	3.5	0.0	NA	–

*Note*: *n*: 15 for each pesticide at each site; NA: mean and standard deviation not calculated because pesticide was detected in only one or no samples; –: maximum concentration absent because pesticide was not detected in any sample.

#### 3.1.2 Neonicotinoid detection frequencies and concentrations

Clothianidin was the most frequently detected neonicotinoid insecticide, present in at least 80% of the samples collected at each site, apart from Site #1 (Table 3). Thiacloprid was not detected in any sample, while imidacloprid and thiamethoxam were detected in 13% and <2% of all the samples over the monitoring period, respectively. No measured concentrations exceeded any freshwater invertebrate chronic toxicity levels (Table 2).

### 3.2 Spatial dynamics

**FIGURE 3:**
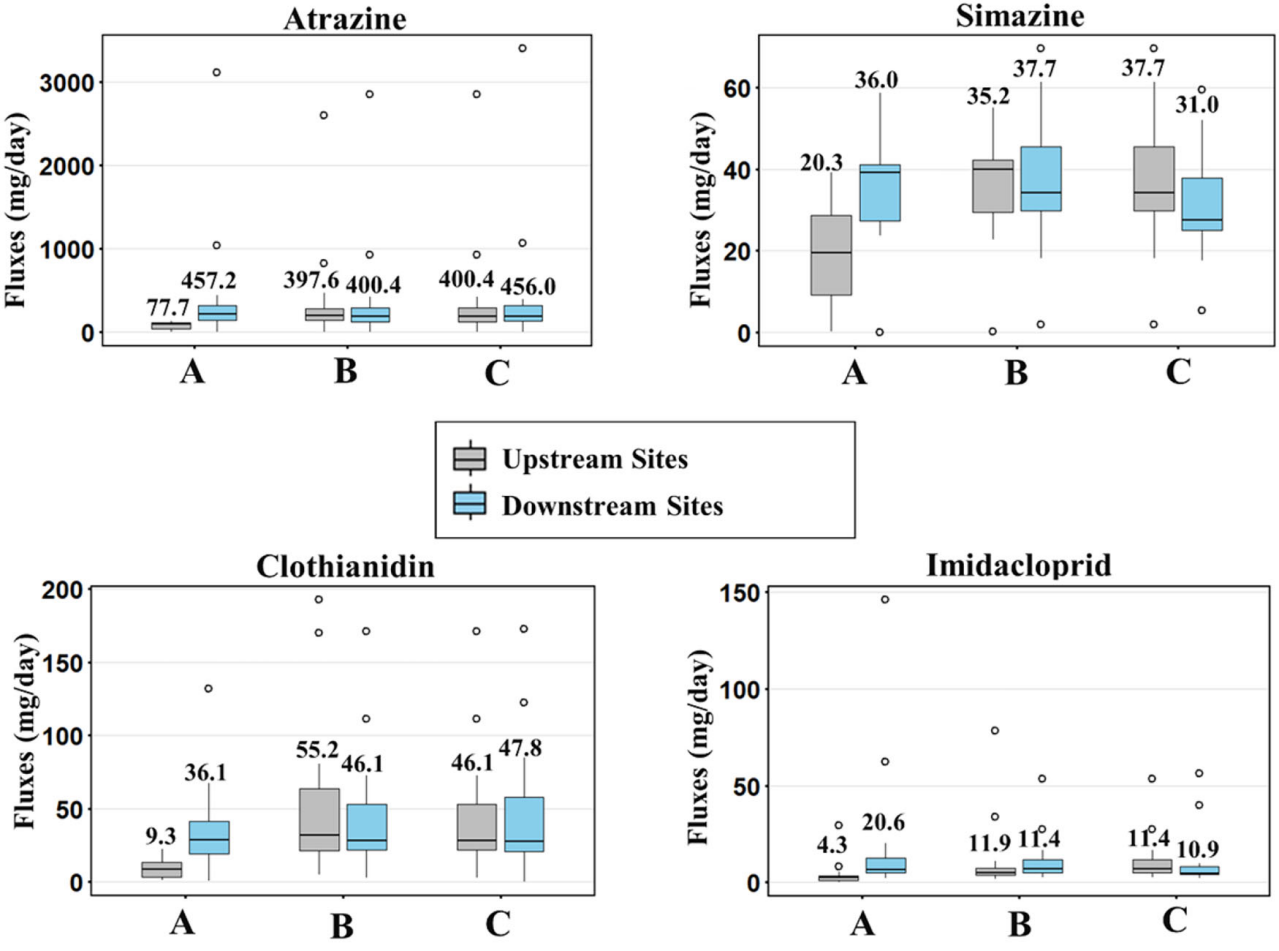
Box plots showing pesticide load distribution at upstream and downstream sites. The upstream and downstream pairs are indicated by the letters on the *x*‐axis: (A) Site #1 (upstream) and Site #2 (downstream), (B) Site #2 and 3 (upstream) and Site #4 (downstream), and (C) Site #4 (upstream) and Site #5 (downstream). The number on each boxplot indicates the mean pesticide flux value. In determining fluxes upstream of Site #4, fluxes for Sites #2 and 3 were averaged.

The one‐sided Wilcoxon signed‐rank test showed that pesticide loads downstream were significantly greater than upstream (*p* < 0.05, Table S4) only between Sites 1 and 2 (Figure 3, Pair A) for all pesticides and between Sites 2 and 3 and 4 (Figure 3, Pair B) for imidacloprid. Mean and median values for all pesticide fluxes were higher at Site #2 (Figure 3), indicating that additional pesticide loads enter the stream at Site #2, likely due to the presence of more pesticide sources within the Site #2 sub‐watershed. For imidacloprid at Pair B, even though downstream fluxes were significantly greater, mean fluxes were higher upstream due to a few high‐magnitude observations (Figure 3). For the other site pairs (*p* > 0.05), the median and mean concentrations were either lower downstream, or there was not a significant increase between the sites (Figure 3). This implies that fluxes remain relatively uniform between upstream and downstream sites or that dilution may be occurring at downstream sites.

Further, even in cases where downstream fluxes were not significantly greater, Spearman's rank correlation showed that upstream and downstream fluxes were positively correlated (*ρ* ≥ 0.6 and *p*‐value < 0.05, Table S4), except for atrazine at Pair A and simazine at Pairs B and C. This indicates that even though downstream fluxes are not always higher than upstream loads, increases in upstream fluxes are generally associated with increases in downstream fluxes. Such headwater streams, comprising largely of first‐, second‐, or third‐order streams, have low dilution capacities, with minimal change between sites (Leibowitz et al., 2018; Lorenz et al., 2017). However, timing of agricultural pesticide applications between the sub‐watersheds is expected to be similar; hence, fluxes at upstream and downstream sites increase simultaneously. Also, apart from the sub‐watersheds for Sites #1 and #2, all the other sub‐watersheds had at least 48% of karst features intersecting agricultural fields (Table S2). Karst features can lead to the rapid movement of pesticides between sub‐watersheds through subsurface flow conduits that may extend across sub‐watershed boundaries, particularly during episodic events, bypassing natural attenuation or treatment by implemented conservation practices (Huang et al., 2021; Reberski et al., 2022; Taylor & Greene, 2008).

### 3.3 Precipitation and pesticide occurrence

Pearson correlation revealed that over the monitoring period and cumulatively across the sites, atrazine showed a weak but statistically significant negative correlation with 3‐day cumulative precipitation (*r* = −0.245 and *p*‐value = 0.043), indicating a pattern of lower concentrations with increasing precipitation consistent with findings by Bachetti et al. (2021). However, when examined on a seasonal basis, the correlations revealed that these relationships changed over the growing season (Table 4). Early in the growing season, atrazine concentrations showed a statistically significant positive correlation to precipitation (*p* < 0.05). A peak in mean concentration across all sites was observed to occur around mid‐May, less than two weeks after the reported start of the planting season in the watershed (Chesapeake Bay Foundation, 2021), followed by a relatively consistent decline (Figure S2) even when precipitation was high. These patterns are characteristic of a “spring flush,” which is known to impact water quality and to be a seasonal stressor to aquatic ecosystems (Stoeckel et al., 2012). The patterns also indicate the role of surface runoff in the transport of atrazine, a surface‐applied pesticide with moderate to high log *K*_oc_ values (Table 2).

**TABLE 4:** Pearson correlation coefficient (*r*) and *p*‐values showing the relationship between mean pesticide concentration across sites for each sampling event and cumulative 3‐day precipitation preceding each sampling event at seasonal levels.

	Atrazine		Simazine		Clothianidin	
	*r*‐value	*p*‐value	*r*‐value	*p*‐value	*r*‐value	*p*‐value
**Spring**	0.702	0.0008	−0.209	0.376	0.635	0.027
**Summer**	−0.342	0.045	−0.594	0.0001	0.210	0.248
**Early fall**	0.417	0.122	0.136	0.628	0.668	0.018

Similar to atrazine, simazine concentrations over the monitoring period showed a negative linear relationship with precipitation (*r* = −0.478 and *p*‐value < 0.0001), with seasonal trends showing a statistically significant negative correlation only in summer (Table 4). However, unlike atrazine, simazine concentration peaked multiple times, with mean concentrations across sites above 3.25 ng/L observed in April, June, and October (Figure S3). These additional observed peaks could suggest multiple simazine application times, potentially due to residential usage of herbicides in the watershed, which are known to impact surface water quality (Hanke et al., 2010; Myers et al., 2022). The peaks also occurred when no recent (i.e., within 3 days) precipitation occurred before sampling, suggesting the influence of shallow lateral flow or groundwater transport of simazine in the watershed.

Clothianidin concentrations over the monitoring period showed a weak, positive linear relationship to precipitation (*r* = 0.336 and *p*‐value = 0.011). All seasonal correlations also had positive correlations, but they were only statistically significant in spring and early fall (*p *< 0.05, Table 4). This pattern suggests that precipitation resulted in the mobilization of clothianidin via surface runoff, which could also be influenced by its high solubility in water (Table 2). Similar patterns were observed in a study in the Great Lakes region (Struger et al., 2017) that reported a positive correlation between clothianidin levels and precipitation recorded a day prior to sampling. Berens et al. (2021) linked precipitation events to high neonicotinoid mobilization in surface runoff into the monitored streams in the Midwestern region. In the current study, mean concentrations across sites were found to peak as high as 9.84 ng/L in July (Figure S4), with subsequent increases in mean concentrations not exceeding 5.75 ng/L even upon significant precipitation, suggesting source‐limited transport dynamics, with most clothianidin mobilized during early precipitation events in the growing season.

### 3.4 Pesticide trends and land use

**FIGURE 4:**
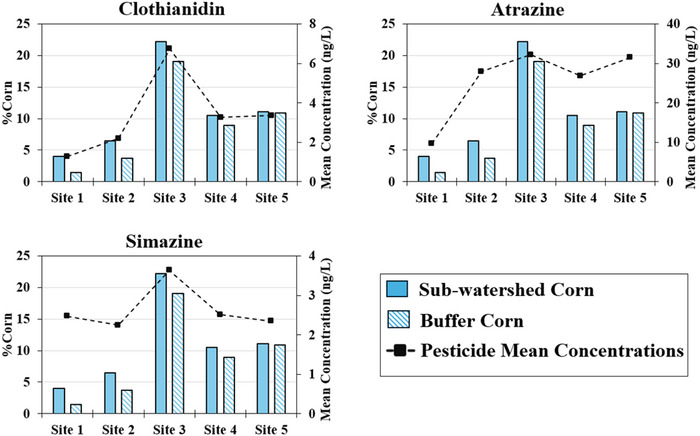
Corn land cover (%) and mean pesticide concentrations (ng/L) across the sub‐watersheds. Bars represent the cumulative corn land cover upstream of each site and their respective buffer zones, while the line indicates the corresponding mean pesticide concentrations.

Even though farm‐level pesticide applications on different crops were not verified, mean clothianidin concentrations fluctuated with sub‐watershed and buffer zone corn percentage (Figure 4). This is consistent with the reported widespread use of clothianidin on corn, as well as studies that link its occurrence in surface water and residues in soil to corn field applications (Hladik et al., 2014; Schaafsma et al., 2015). However, for atrazine, despite mean concentration being highest at Site #3 with the most corn land cover, the sub‐watershed for Site #2, which had a lower percentage of land in corn, still exhibited a high mean concentration (Figure 4). This indicates that there could be other sources of atrazine within Site #2 sub‐watershed, either domestic or urban, with the sub‐watershed having the largest area in residential land use (20.9%, Table 1). Despite the highest mean concentrations at Site #3, simazine did not show clear influence from corn land cover, given less variation in concentration across the sites (Figure 4).

### 3.5 *C*–*Q* relationship

**FIGURE 5:**
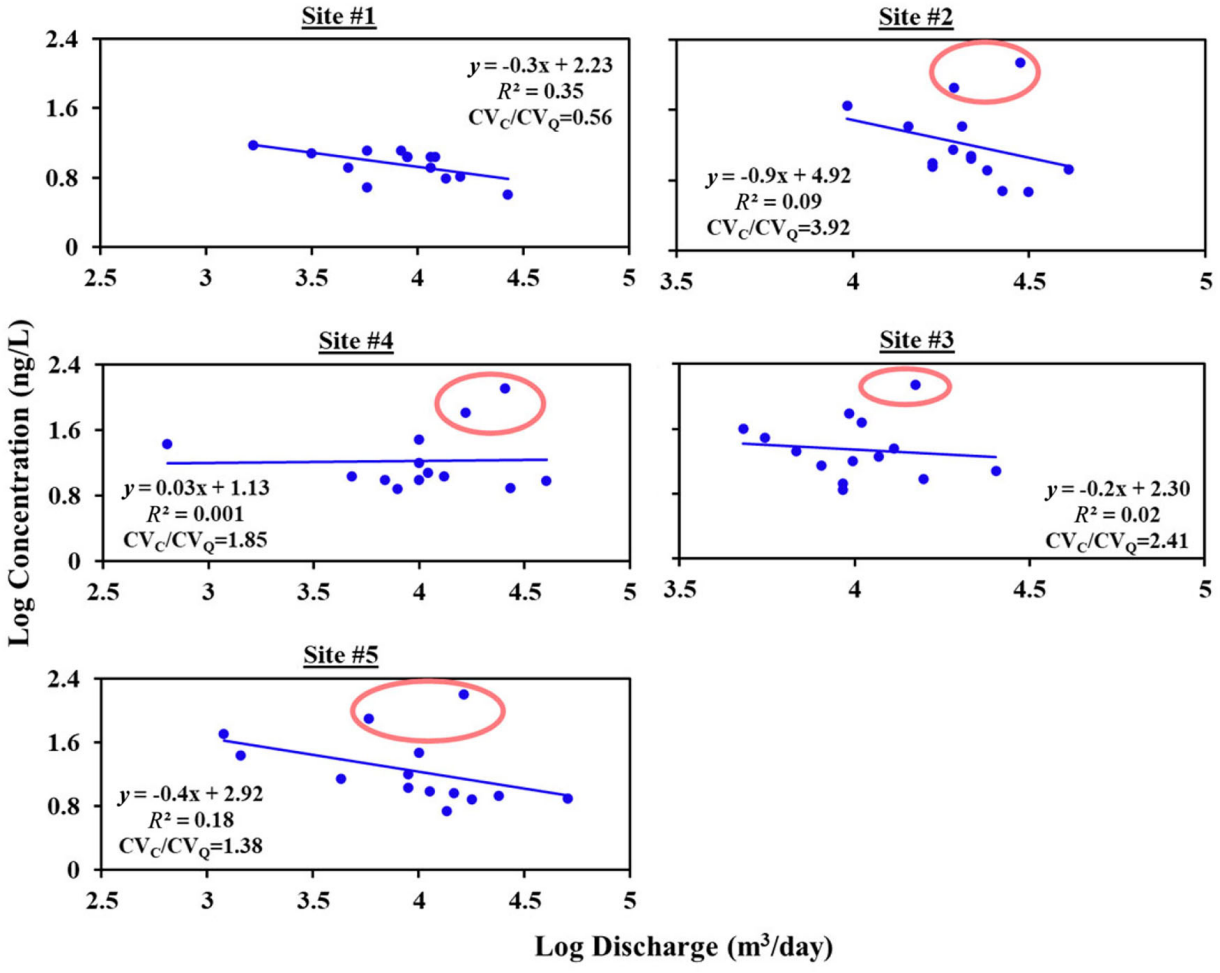
Concentration‐discharge (C‐Q) relationships for atrazine at each monitoring site. Circled points indicate the highest concentrations recorded during the spring season, which drive the observed concentration variabilities at the sites.

Across all sites except Site #1, atrazine had a CV_C_/CV_Q_ ratio greater than 1 (Table 5), indicating a chemodynamic export regime and a likely influence of episodic events such as precipitation early in the growing season, driving the high variability in concentrations. The relatively stable concentrations at Site #1 (CV_C_/CV_Q_ = 0.56) compared to other sites could be attributed to it being the most upstream site, with lower corn land cover relative to the rest of the watershed. Site #2 showed the highest concentration variability with respect to discharge (CV_C_/CV_Q _= 3.92), with the highest atrazine concentrations (140 ng/L and 72 ng/L, Figure 5) recorded during the spring season. This is consistent with Stoeckel et al. (2012) finding that atrazine concentrations increased with an increase in discharge during storm events after the planting season in May and June. Therefore, atrazine transport in surface runoff could be dominant, either in the dissolved phase or bound to sediment (Taghavi et al., 2011). However, despite the increase in atrazine loads during a few episodic events (shown in red circles in Figure 5), all the sites showed a dilution pattern with discharge (*b* < 0 – Table 5) except at Site #4, which exhibited a weak accretion pattern with discharge (Figure 5). This implies a dilution‐driven chemodynamic behavior at sites with CV_C_/CV_Q _> 1 and *b* < 0.

**TABLE 5:** Pesticide concentration and discharge coefficient of variation ratios (CV_C_/CV_Q_) and *b* values (slope of the concentration‐discharge [C‐Q] linear relationship) by site.

Pesticide	Site	CV_C_/CV_Q_	*b*‐value
**Atrazine**	1	0.56	−0.3
	2	3.92	−0.9
	3	2.41	−0.2
	4	1.85	0.03
	5	1.38	−0.4
**Simazine**	1	0.61	−0.3
	2	0.94	−0.6
	3	0.57	−0.5
	4	0.28	−0.1
	5	0.29	−0.1
**Clothianidin**	1	NA	NA
	2	1.12	0.6
	3	2.08	0.4
	4	0.81	0.2
	5	0.62	0.2

Simazine had a CV_C_/CV_Q_ ratio <1 across all sites (Table 5), indicating that the transport dynamics could be categorized as chemostatic to moderately chemodynamic behavior (Thompson et al., 2011). All the sites had negative *b* values (Table 5; Figure S5), implying that simazine concentrations decrease during high flow events. This pattern suggests that compared to atrazine, simazine sources are largely protected from the influences of episodic surface runoff events. As a result, simazine is likely transported predominantly in groundwater, leading to dilution as discharge increases.

Clothianidin detection frequency at Site #1 was low (Table 3), and therefore a *C‐Q* relationship was not determined for the site. The remaining sites had positive *b* values (Table 5; Figure S6), indicating an accretion transport pattern where clothianidin was mobilized with increases in discharge. Kibuye et al. (2020) investigated emerging organic contaminants and observed an accretion pattern for another neonicotinoid, thiamethoxam, linking it to surface runoff transport. However, since it is introduced to soil below the surface, it can also be transported in subsurface flow and delivered to the streams through subsurface drainage (Frame et al., 2021; Schaafsma et al., 2019). The CV_C_/CV_Q_ values between 0.62 and 2.08 indicate chemodynamic or moderate chemodynamic behavior at the sites, indicating the influence of such episodic events. Bieroza et al. (2018) note that the observed positive response to discharge could mean that clothianidin movement into the stream is transport‐limited, whereby appropriate conditions, such as precipitation generating enough runoff for mobilization, modulate transport. However, given that clothianidin was found to be less sensitive to precipitation later in the growing season (Figure S3, see Section 3.3), export dynamics could be transitioning from transport limited to source limited over the growing season.

The different transport patterns observed for the contaminants could also be explained by their properties and the influence of karst features within agricultural fields (Table S2). Simazine is less soluble in water compared to atrazine and clothianidin (Table 2) and more likely to bind to sediments (Glenn & Angle, 1987). During storm events, surface depressions could collect surface runoff and shallow lateral flow temporarily before transitioning the flow into overland flow (Meng et al., 2021) or acting as recharge points if connected to deeper karst flow paths (Somaratne, 2014). Atrazine and clothianidin, which are likely present largely in the dissolved phase, can then be remobilized in surface runoff or move through groundwater into streams in short travel times without any attenuation (White, 1988), thereby maintaining the influence of episodic events.

However, sediment‐bound contaminants could collect in the depressions. Given the diverse transport pathways of water in karst features (White, 2018), sediment‐bound contaminants could be subject to slow rather than rapid transport in groundwater (Vadillo & Ojeda, 2022). In this case, surface depressions could act as temporary sinks of solutes in sediments with post‐rainfall event release into groundwater through diffuse flow in soil matrix (Field, 1992; Ghasemizadeh et al., 2012). If the depressions are connected to karst flow paths, sediment clogging may occur, creating immobile zones, which could act as points of solute storage and gradual diffusion back to mobile zones (Field & Pinsky, 2000; Shapiro et al., 2008). This may also explain the observed peaks in simazine concentrations during baseflow (Figure S3; Section 3.3) and relatively stable concentrations across the seasons (Table S3). Unlike atrazine and clothianidin, simazine may remain trapped within the surface depressions or karst flow paths for longer periods, which may also contribute to its attenuation (Brad et al., 2022; Campanale et al., 2022).

### 3.6 Limitations

Even though we have observed karst features (e.g., disappearing streams and surface depressions) on the fields we had access to, we did not attempt to comprehensively verify the karst features within the watershed shown in Figure 1. Karst features can remain stable or transform over time whereby, depending on environmental or geomorphic factors, features such as surface depressions could be filled or become more defined (e.g., transform into sinkholes) (Veress, 2022). Additionally, we did not verify pesticide applications but rather made inferences based on typical applications on corn and information from central Pennsylvanian farmers.

## 4 CONCLUSIONS AND IMPLICATIONS

Overall, atrazine, simazine, and clothianidin were the most frequently detected pesticides, reflecting their widespread use across the watershed. Transport analyses revealed compound‐specific pathways, with simazine largely transported in groundwater, while atrazine and clothianidin were predominantly transported in surface runoff and therefore more likely to be mitigated by management practices targeting overland flow. The biweekly sampling regime captured the seasonal “spring flush,” highlighting the importance of high frequency or post‐storm sampling to inform riparian buffer placement and accurately evaluate downstream water quality responses. The nested watershed approach revealed both site‐specific occurrence and transport patterns, as well as cumulative downstream effects. A high degree of hydrological connectivity due to the prevalence of karst features, combined with dispersed agricultural land use across the watershed, enabled upstream pesticide applications to persist and influence downstream concentrations. These results suggest that effective implementation of conservation practices in karst systems should consider the role of surface depressions and karst flow pathways in accelerating contaminant conveyance to surface water and likely bypassing riparian buffers. Conservation strategies in these regions should ensure that riparian buffers are placed where they can intercept surface runoff pathways and that buffer management is integrated with upgradient, field‐based practices that reduce pesticide application and mitigate mobilization through karst features.

## AUTHOR CONTRIBUTIONS

**Henry J. Kibuye**: Conceptualization; data curation; formal analysis; investigation; methodology; validation; visualization; writing—original draft. **Tamie L. Veith**: Conceptualization; investigation; methodology; resources; supervision; writing—review and editing. **Tyler A. Groh**: Conceptualization; funding acquisition; methodology; supervision; writing—review and editing. **Heather E. Preisendanz**: Conceptualization; funding acquisition; investigation; methodology; project administration; resources; supervision; validation; writing—review and editing.

## CONFLICT OF INTEREST STATEMENT

The authors declare no conflicts of interest.

## Supporting information



The supplemental materials include additional details regarding sample processing and analysis along with data and figures that expand the summarizations in the main manuscript.
