# Supplementary material for: Neonicotinoid and s‐triazine pesticide transport dynamics in a small karst agricultural watershed
Source: J Environ Qual. 2026 Feb 23;55(2):e70155. doi: 10.1002/jeq2.70155 (PMC12929202; doi:10.1002/jeq2.70155)
Supplement: Supplementary file 1 — The supplemental materials include additional details regarding sample processing and analysis along with data and figures that expand the summarizations in the main manuscript. [file JEQ2-55-0-s001.docx]

**Supplemental Material: Neonicotinoid and s-triazine pesticide transport dynamics in a small karst agricultural watershed**

Henry J. Kibuye^1^, Tamie L. Veith^2^, Tyler A. Groh^3^, and Heather E. Preisendanz^1,4*^

^1^Department of Agricultural & Biological Engineering, The Pennsylvania State University, University Park, PA 16802

^2^USDA Agricultural Research Service, Pasture Systems and Watershed Management Research Unit, University Park, PA 16802

^3^Department of Ecosystem Science and Management, The Pennsylvania State University, University Park, PA 16802

^4^Institute for Sustainable Agricultural, Food, and Environmental Science, College of Agricultural Sciences, The Pennsylvania State University, University Park, PA 16802

* Corresponding Author: hpreisen@psu.edu.

13 pages total: 1 page of text, 6 figures, 4 tables.

**Table of Contents**

Text: Sample Processing and Analysis 2

List of Figures 3

List of Tables 10

**Sample Processing and Analysis**

Samples were filtered through 0.47 μm glass fiber filter paper before extraction using solid phase extraction (SPE) cartridges containing a hydrophilic-lipophilic-balanced (HLB), water-wettable, reversed-phase universal sorbent for acidic, neutral, and basic compounds (Oasis® HLB). Once extracted, samples were eluted using 8 mL of ultra-high performance liquid chromatography coupled with mass spectrometry (UHPLC-MS) grade methanol and concentrated to ~1 mL under a gentle stream of nitrogen gas at 60 °C using a nitrogen blowdown evaporator. After each concentrated sample was reconstituted and mixed, 1000 µL of sample was transferred into an amber glass autosampler vial for analysis.

The target analytes were analyzed and quantified using a high-resolution accurate mass (HRAM) Q Exactive mass spectrometer (Thermo-Fisher Scientific, Bremen, Germany), interfaced with an ICS-5000+ chromatography system (Thermo-Fisher Dionex, Sunnyvale, CA) via a heated electrospray injection (HESI) source. 10 µL of sample was injected onto a 2.1 x 100 mm, 2.6 µm Accucore™ Phenyl Hexyl column (Thermo-Fisher Scientific, USA) and eluted using 0.1% formic acid in water (A), and acetonitrile and 0.1% formic acid (B). The initial mobile phase composition was set at 98% A:2% B at 0 minutes, held for two minutes followed by a linear gradient from 2% B to 100% B in eight minutes then held for two minutes and back to 2% B and kept constant for two minutes. The flow rate during elution was 0.4 mL/min and a total runtime of 14 minutes. The mass range of the mass spectrometer was 65 to 750 m/z with a resolution of 70,000 and operated in data dependent MS2 (ddMS2) mode for all analytes in the predefined list. The ddMS2 mode used a resolution of 17,500 and used normalized collision energies of 10, 30, and 60 eV.

**List of Figures**

Figure S1. Flow duration curves for the monitoring period and stage-derived instantaneous discharge at the time of sampling for the most upstream (Site 1) and most downstream (Site 5) sites, illustrating the range of flow conditions captured during sampling. Deviation of instantaneous discharge from the flow duration curve indicates sub-daily discharge variation. 4

Figure S2. Atrazine: (Top) Time series data for average grab sample concentrations across the sampling sites, instantaneous discharge, and average daily discharge. Discharge measurements are taken from the most downstream site (Site 5). (Bottom) Average grab sample concentrations across the sampling sites and cumulative precipitation in the three days prior to sampling. 5

Figure S3. Simazine: (Top) Time series data for average grab sample concentrations across the sampling sites, instantaneous discharge, and average daily discharge. Discharge measurements are taken from the most downstream site (Site 5). (Bottom) Average grab sample concentrations across the sampling sites and cumulative precipitation in the three days prior to sampling. 6

Figure S4. Clothianidin: (Top) Time series data for average grab sample concentrations across the sampling sites, instantaneous discharge, and average daily discharge. Discharge measurements are taken from the most downstream site (Site 5). (Bottom) Average grab sample concentrations across the sampling sites and cumulative precipitation in the three days prior to sampling. 7

Figure S5. Simazine: Concentration-discharge relationships at the sampling sites. 8

Figure S6. Clothianidin: Concentration-discharge relationships at the sampling sites. 9


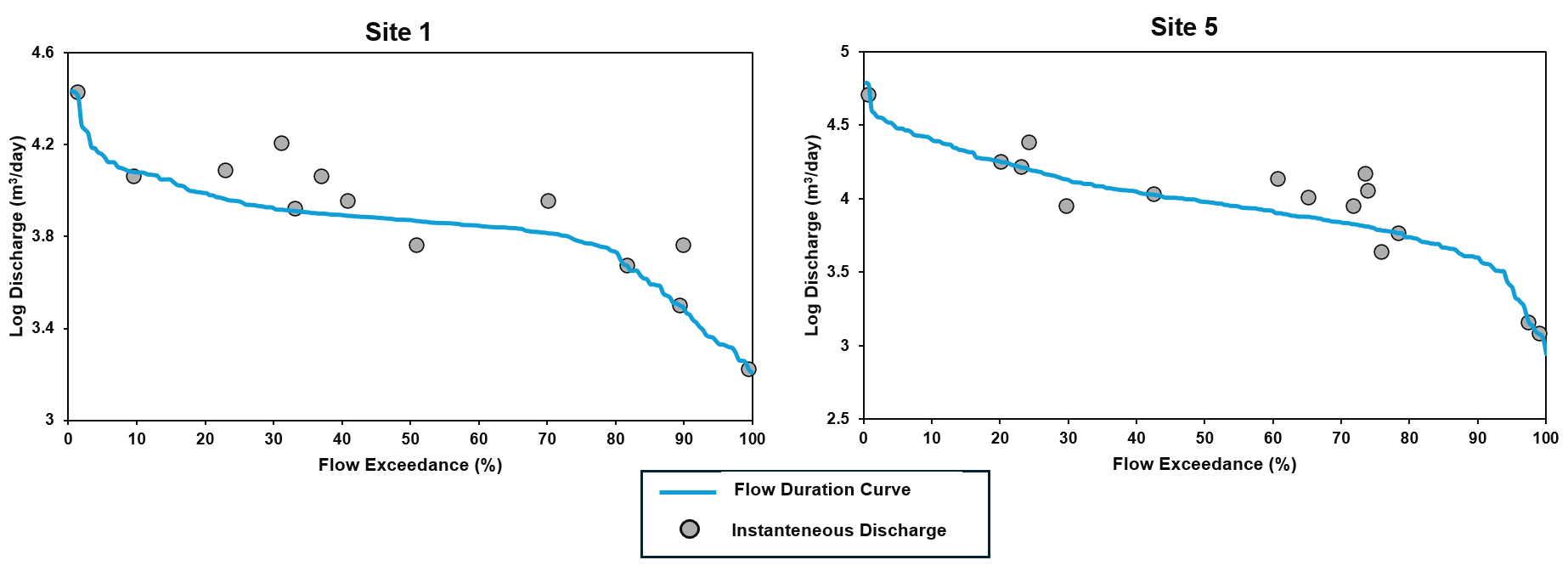


Figure S1. Flow duration curves for the monitoring period and stage-derived instantaneous discharge at the time of sampling for the most upstream (Site 1) and most downstream (Site 5) sites, illustrating the range of flow conditions captured during sampling. Deviation of instantaneous discharge from the flow duration curve indicates sub-daily discharge variation.


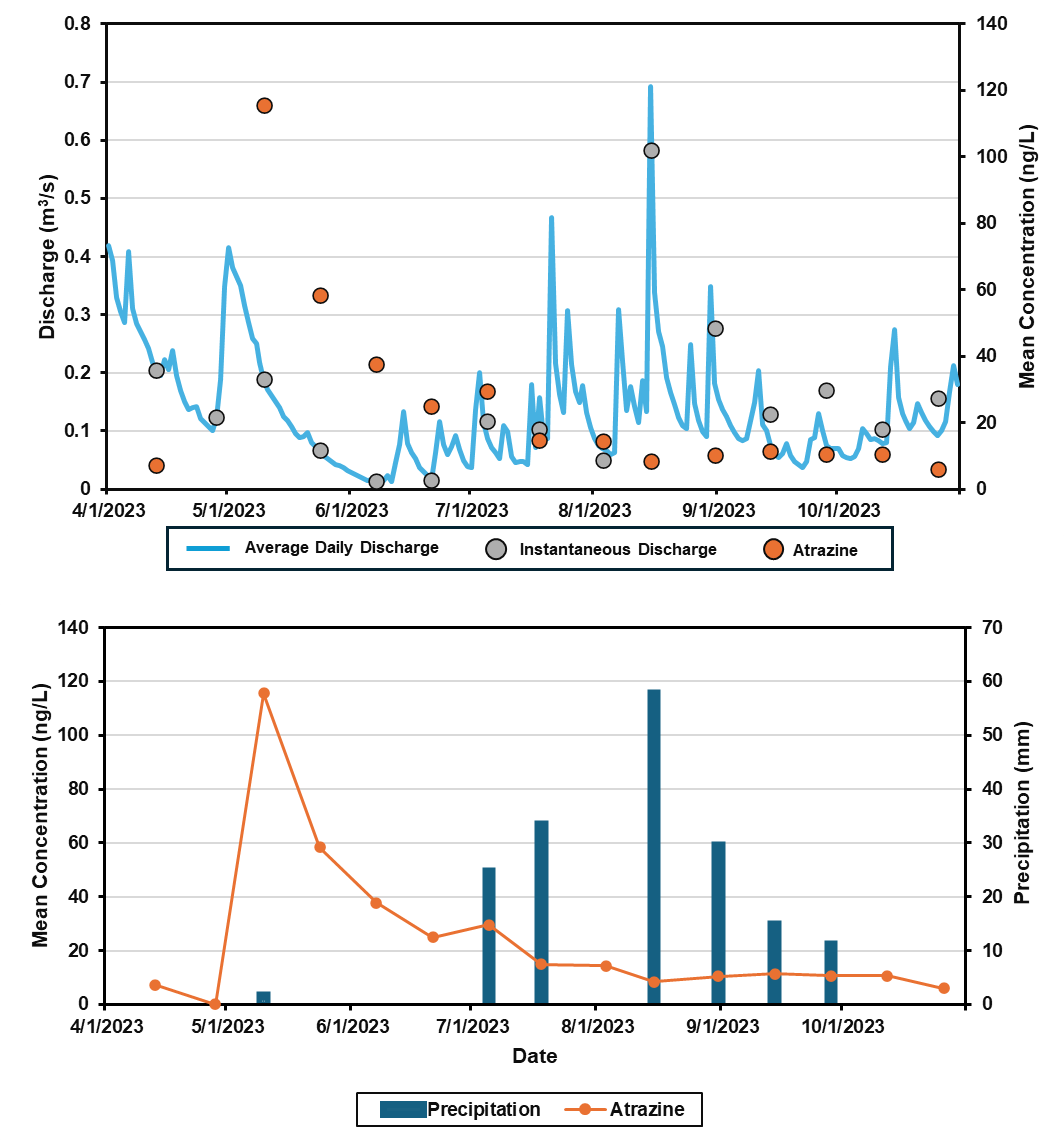
Figure S2. Atrazine: (Top) Time series data for average grab sample concentrations across the sampling sites, instantaneous discharge, and average daily discharge. Discharge measurements are taken from the most downstream site (Site 5). (Bottom) Average grab sample concentrations across the sampling sites and cumulative precipitation in the three days prior to sampling.


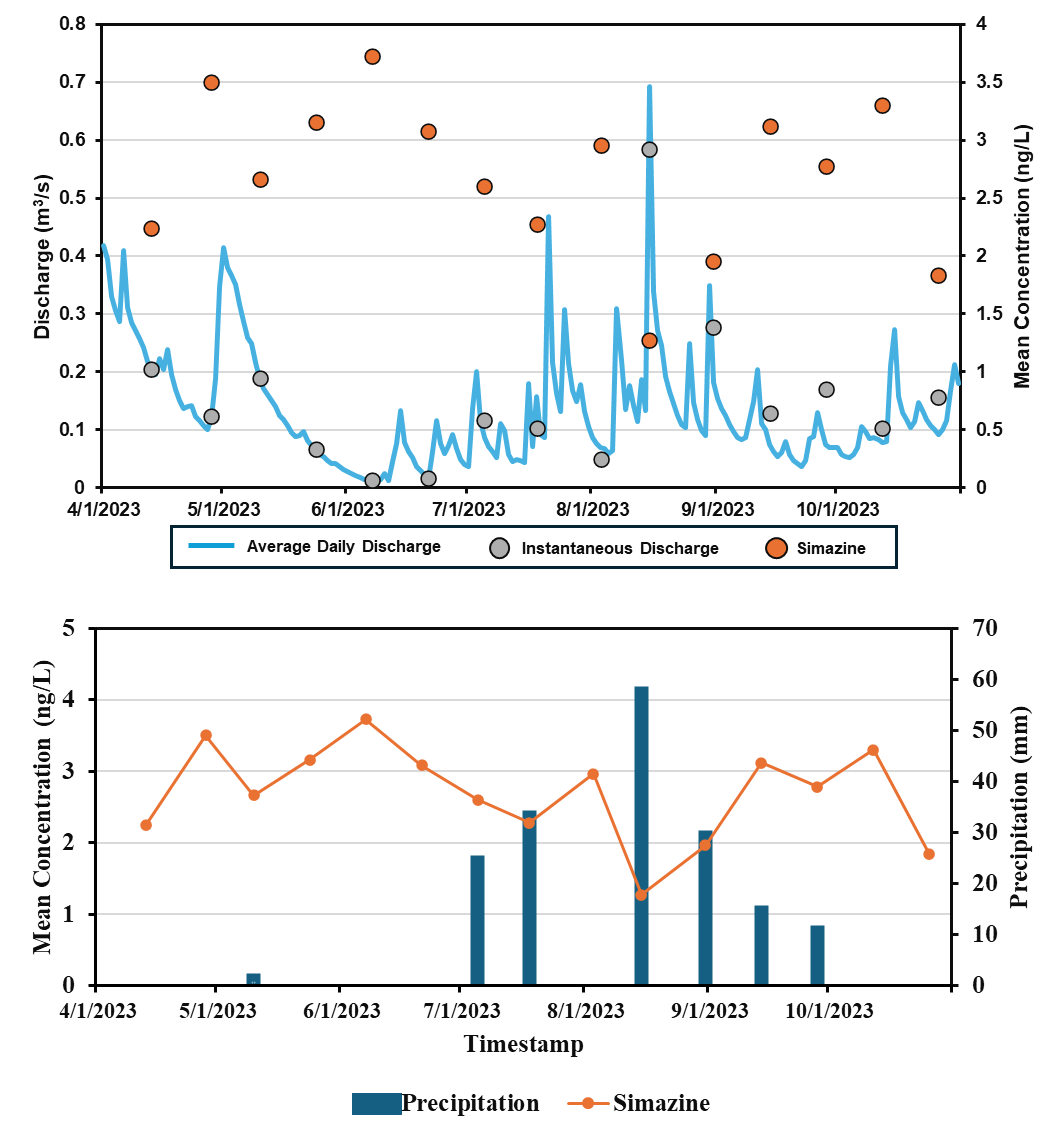


Figure S3. Simazine: (Top) Time series data for average grab sample concentrations across the sampling sites, instantaneous discharge, and average daily discharge. Discharge measurements are taken from the most downstream site (Site 5). (Bottom) Average grab sample concentrations across the sampling sites and cumulative precipitation in the three days prior to sampling.


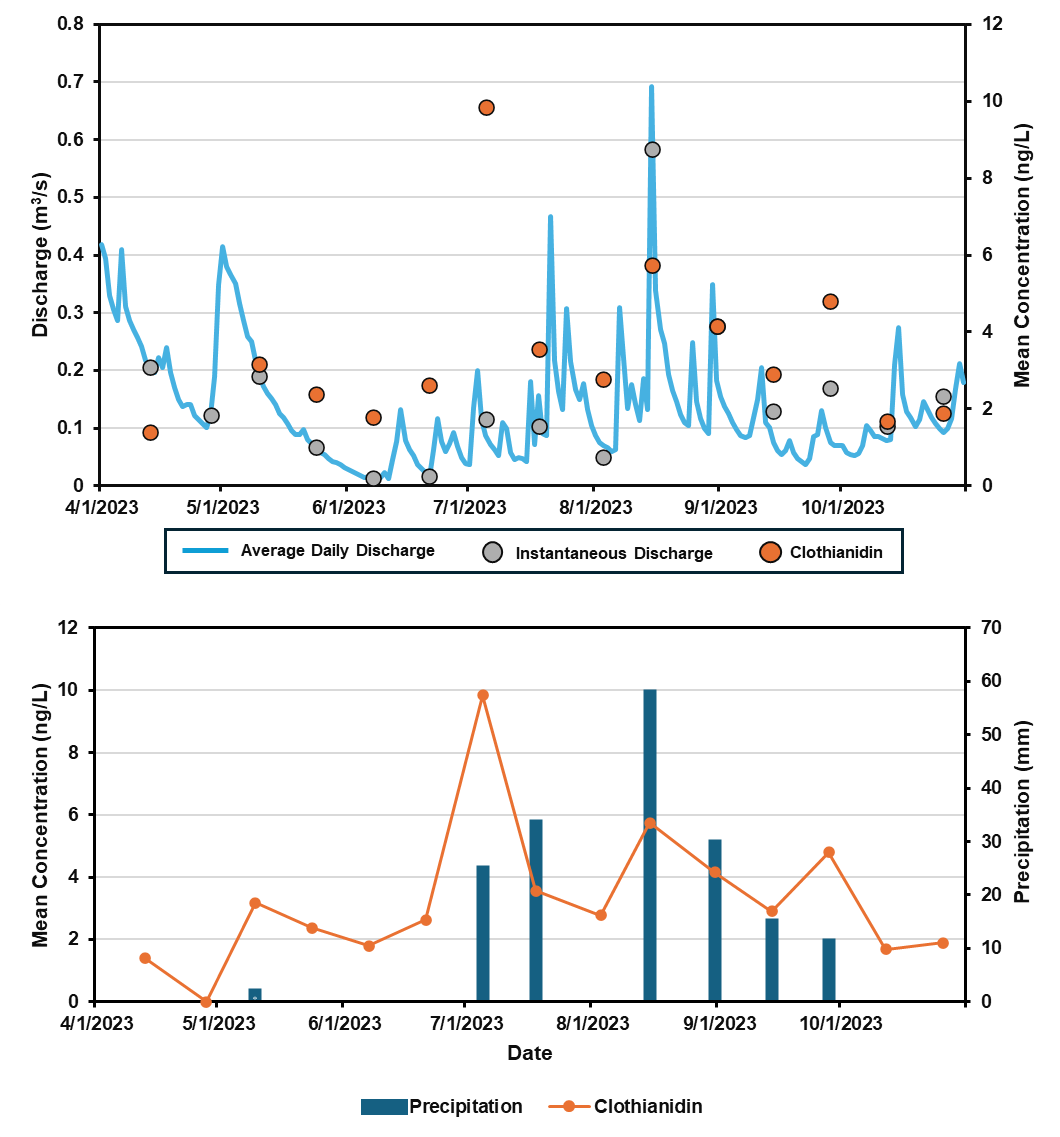


Figure S4. Clothianidin: (Top) Time series data for average grab sample concentrations across the sampling sites, instantaneous discharge, and average daily discharge. Discharge measurements are taken from the most downstream site (Site 5). (Bottom) Average grab sample concentrations across the sampling sites and cumulative precipitation in the three days prior to sampling.


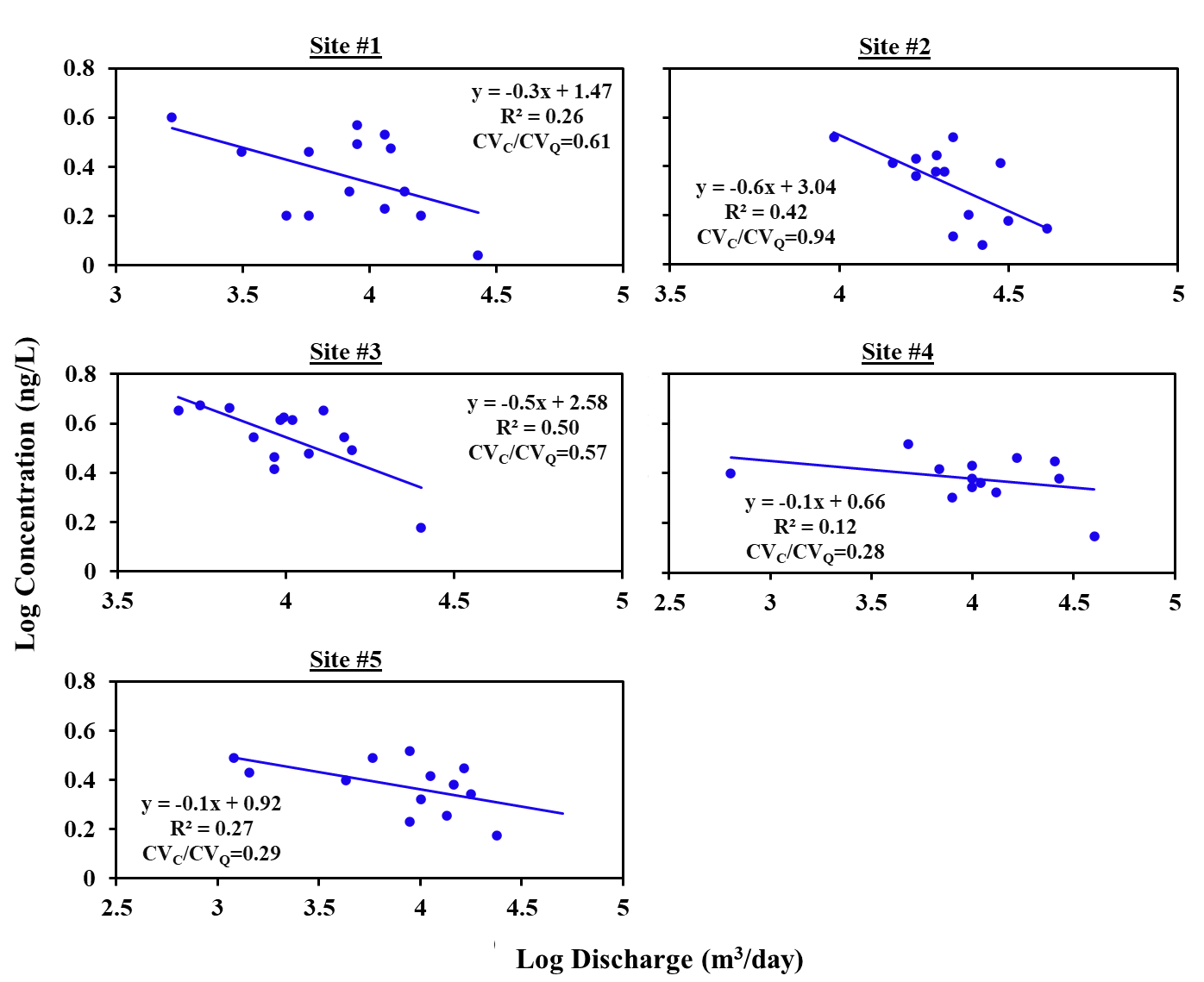


Figure S5. Simazine: Concentration-discharge relationships at the sampling sites.


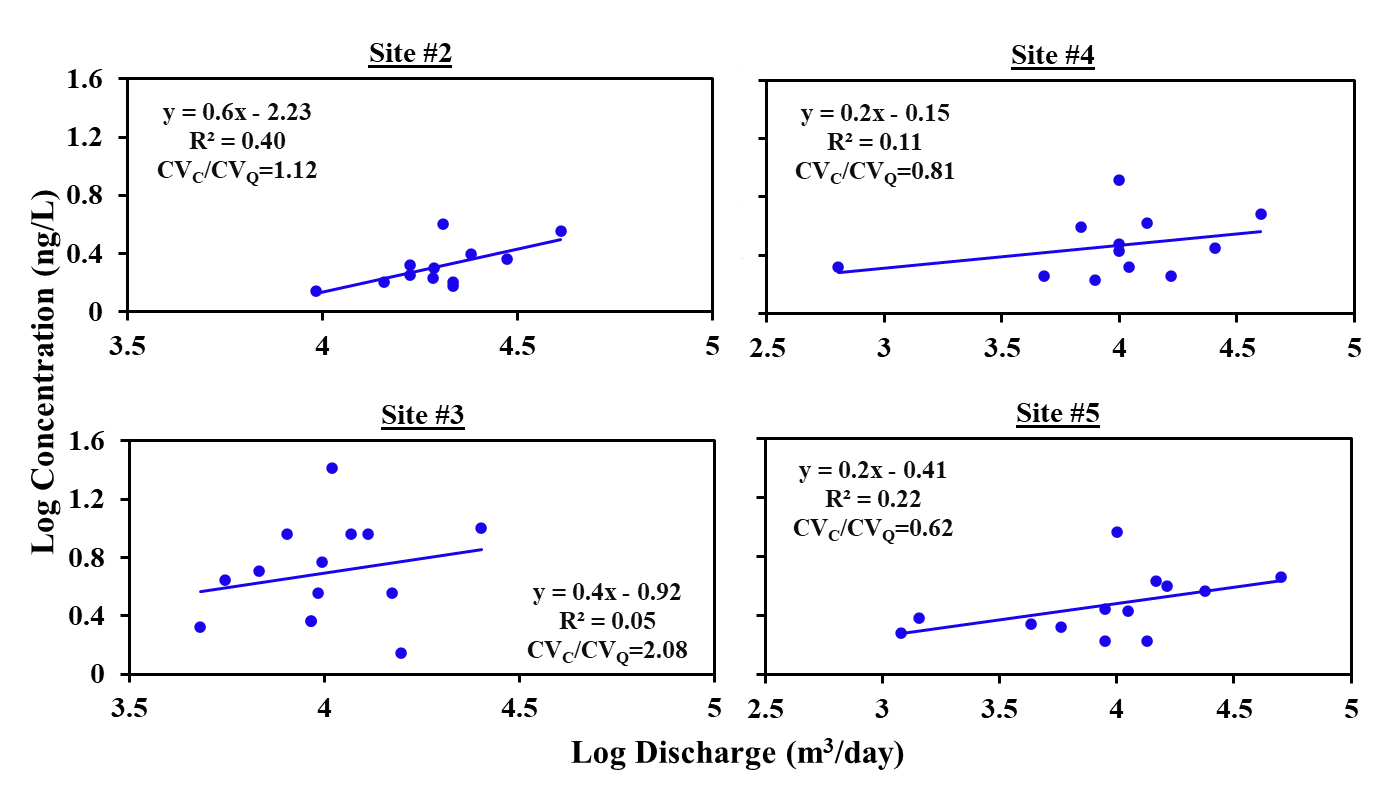


Figure S6. Clothianidin: Concentration-discharge relationships at the sampling sites.

**List of Tables**

Table S1. Soil series and textures and their percentage within the watershed (NRCS, 2024). 11

Table S2. Summary of karst feature distribution across the sub-watersheds, including the number and percentage of karst features intersecting agricultural fields and the proportions of different karst feature types within the agricultural fields. 12

Table S3. Seasonal mean pesticide concentrations (ng/L) across monitoring sites. 13

Table S4. Site-based analysis of upstream and downstream pesticide fluxes showing one-sided Wilcoxon signed-rank test *p*-value, spearman’s correlation rho (ρ) and *p*-value, and paired fluxes (n). The Upstream Site flux of Site #4 is the average of the Site #2 & Site #3 fluxes. 13

**Table S1.** Soil series and textures and their percentage within the watershed (NRCS, 2024).

| Soil Series and Textures | Percentage |
| --- | --- |
| Morrison sandy loam & very stony sandy loam | 40.05 |
| Hublersburg silt loam | 12.49 |
| Hagerstown silt loam & silty clay loam | 10.77 |
| Murrill gravelly loam, channery silt loam & very stony silt loam | 6.50 |
| Hazleton channery sandy loam & extremely stony sandy loam | 5.65 |
| Opequon clay loam, Hagerstown complex & rock outcrop complex | 4.51 |
| Laidig channery loam & extremely stony loam | 4.33 |
| Andover channery silt loam & very stony loam | 3.17 |
| Ungers channery & very stony loam | 2.39 |
| Buchanan channery loam | 1.58 |
| Melvin silt loam | 1.11 |
| Wyoming gravelly sandy loam | 1.04 |
| Nolin silt loam | 0.87 |
| Tyler silt loam | 0.80 |
| Chagrin soils | 0.67 |
| Millheim silt loam | 0.64 |
| Lindside soils | 0.61 |
| Berks channery silt loam | 0.48 |
| Leetonia sand | 0.34 |
| Newark silt loam | 0.24 |
| Clarksburg silt loam | 0.17 |
| Vanderlip loamy sand | 0.14 |
| Albrights silt loam | 0.12 |
| Urban-land Hagerstown | 0.12 |
| Weikert shaly silt loam | 0.10 |
| Berks and Weikert soils | 0.07 |
| Barbour soils | 0.04 |
| Ernest channery silt loam & very stony silt loam | 0.03 |
| Wharton silt loam | 0.02 |
| Dunning silty clay loam | 0.02 |
| Monongahela silt loam | 0.02 |

**Table S2.** Summary of karst feature distribution across the sub-watersheds, including the number and percentage of karst features intersecting agricultural fields and the proportions of different karst feature types within the agricultural fields

|  |  |  | Karst types in Ag fields (%) | | |
| --- | --- | --- | --- | --- | --- |
| Sub-Watershed | **Total *n*** | ***n* (%) in Ag fields** | **Caves** | **Sinkholes** | **Surface Depressions** |
| 1 | 131 | 27 (21) | 0.00 | 0.00 | 100.00 |
| 2 | 708 | 169 (24) | 0.59 | 3.55 | 95.86 |
| 3 | 239 | 114 (48) | 0.00 | 4.39 | 95.61 |
| 4 | 659 | 334 (51) | 0.00 | 1.20 | 98.80 |
| 5 | 122 | 86 (70) | 0.00 | 6.98 | 93.02 |

**Table S3.** Seasonal mean pesticide concentrations (ng/L) across monitoring sites.

| Season | Atrazine | Simazine | Clothianidin | Imidacloprid |
| --- | --- | --- | --- | --- |
| Spring | 55.7 | 2.9 | 2.4 | NA |
| Summer | 16.3 | 2.5 | 4.6 | 2.5 |
| Early Fall | 9.11 | 2.6 | 2.8 | 2.5 |

**Table S4.** Site-based analysis of upstream and downstream pesticide fluxes showing one-sided Wilcoxon signed-rank test *p*-value, spearman’s correlation rho (ρ) and *p*-value, and paired fluxes (n). The Upstream Site flux of Site #4 is the average of the Site #2 & Site #3 fluxes.

| Pesticide | Upstream Site | Downstream Site |  | Wilcoxon |  | Spearman | |
| --- | --- | --- | --- | --- | --- | --- | --- |
|  |  |  |  | *p*-value |  | ρ (rho) | *p*-value |
|  | Site 1 | Site 2 |  | < 0.001 |  | 0.1 | 0.771 |
| Atrazine | Site 2 & 3 | Site 4 |  | 0.577 |  | 0.8 | 0.002 |
|  | Site 4 | Site 5 |  | 0.773 |  | 0.8 | 0.002 |
|  | Site 1 | Site 2 |  | < 0.001 |  | 0.6 | 0.036 |
| Simazine | Site 2 & 3 | Site 4 |  | 0.719 |  | 0.5 | 0.051 |
|  | Site 4 | Site 5 |  | 0.997 |  | 0.5 | 0.051 |
|  | Site 1 | Site 2 |  | < 0.001 |  | 0.6 | 0.012 |
| Clothianidin | Site 2 & 3 | Site 4 |  | 0.976 |  | 0.9 | < 0.001 |
|  | Site 4 | Site 5 |  | 0.300 |  | 0.9 | < 0.001 |
|  | Site 1 | Site 2 |  | < 0.001 |  | 0.8 | 0.001 |
| Imidacloprid | Site 2 & 3 | Site 4 |  | 0.018 |  | 0.8 | 0.001 |
|  | Site 4 | Site 5 |  | 0.805 |  | 0.7 | 0.005 |
